# Mycological and Bacteriological Quality and Safety of Bottled Water in Ethiopia

**DOI:** 10.2174/1874285801812010200

**Published:** 2018-06-29

**Authors:** Tesfaye L. Bedada, Firehiwot A. Dera, Redwan M. Edicho, Samson G. Gebre, Yosef B. Asefa, Waktole G. Sima, Rahel F. Maheder, Tigist Y. Negassi, Almaz G. Biegna

**Affiliations:** 1 Public Health Microbiology Research Team, Ethiopian Public Health Institute, Addis Ababa, Ethiopia; 2 Nutrition Research Team, Ethiopian Public Health Institute, Addis Ababa, Ethiopia

**Keywords:** Bottled water, Bacteriological, Coliforms, Mycological, Water Quality, Plate Count

## Abstract

**Background:**

Safe water supply is vital and can result in significant benefits to health. However, contaminated bottled water poses a great health risk due to the poor microbiological quality of water.

**Methods and Materials:**

A retrospective study was conducted on 222 Bottled water samples collected from various regions of Ethiopia from January 2008 to December 2015, tested and recorded in Ethiopian Public Health Institute to determine heterotrophic plate count and *Staphylococcus aureus* by pour plate method; for coliforms using multiple tubes fermentation techniques; for mould and yeast count using spread method, and for *Salmonellae* and *Shigella* spp. using ES ISO 6579 and ES ISO 21567. The data was analyzed using SPSS 20 statistical package.

**Results:**

Among the total samples examined from 44 brands, detections of heterotrophic plate count, mould, yeast, total and thermotolerant coliforms, *Escherichia coli* and *Staphylococcus aureus* were observed in 114 (51.4%), 33 (14.9%), 5 (2.3%), 2 (0.9%), 1 (0.5%), 1 (0.5%) and 1 (0.5%) samples respectively, but there were no detections of *Salmonellae* nor *Shigellae* species.

**Conclusion:**

About 40% of bottled water samples were mycologically and bacteriologically unsafe for human consumption. To prevent public health hazards, regular monitoring of bottled water using quality indicators should be a priority agenda.

## 1 INTRODUCTION

Safe water supply is vital and can result in considerable benefits to health [1]. Bottled drinking water is obtained from wells, boreholes, natural springs, municipal water or other sources which are considered to be safe [2]. However, the microbiological quality of bottled water has been questioned over the years [3]. The effectiveness of ozone residue for bottled water disinfection depends on temperature and storage time [4]. Bottled drinking water may contain carbon dioxide that will restrict regrowth potential, but typically no long-lasting disinfectant residual is present [5]. An increase in the demand for bottled water may lead to poor production because of financial interests [6]. Under these improper conditions of bottled water, microbes that can pass through the filters [7] or those introduced during manufacturing and packaging processes [8] can grow up to harmful levels [9] due to additional growth substrates released from bottling materials during storage [7]. Since the bottled water is a ready-to-consume commodity and is consumed in large volumes, the risk of ingesting large numbers of microorganisms is rather high [8]. Such contaminated waters pose great health risk especially when it is used by elderly persons, infants, hospitalized patients and immuno-compromised individuals [10]. Different gastrointestinal diseases are the most common consequences of consuming contaminated drinks [11].

Microorganisms that have been detected in bottled drinking may pose a significant risk to humans. Microbial safety and quality of bottled drinking water of different studies show high contaminations of heterotrophic plate count (HPC), fungal spoilage [12] and fecal indicators [5] and detections of *Staphyclococus aureus* [13], *Shigella* spp [14]. and *Salmonella* spp [15].

The presence of heterotrophic bacteria in large numbers may cause opportunistic infections in individuals who have a weakened immune system [5]. Drug-resistant heterotrophic bacteria can act as reservoirs of resistance plasmids that can freely exchange with pathogenic bacteria in the gastrointestinal tract [8].

Some species of fungi that contained in bottled drinking water can cause illness when they grow using chemicals such as polyethylene terephthalate from which the bottles are made are leached to bottled water and serve as their nutrients. The disease can occur in humans if the fungi produce mycotoxins. These metabolites are toxic and can induce tumours in several animal models [16]. Coliforms imply the possibility of pathogenic enteric microbes presence including *Salmonella* spp., *Shigella* spp. and *Vibrio cholera* [17].

The main objective of the current study was to analyse the microbial quality and safety of bottled drinking water in Ethiopia and to determine their compliance with the standards and guidelines and as well as to see the correlation of fungi with bacterial indicators.

## 2 METHODS AND MATERIALS

### 2.1 Study Design and Period

The study was a obtained retrospective data from test results of bottled drinking water records, which was kept in Public Health Microbiology Research Team laboratory of Ethiopian Public Health Institute from January 2008 to December 2015 to determine the concentrations of hetrotrophic plate count [18] and *Staphylococcus aureus* by pour plate method [19]; for coliforms using multiple tubes fermentation techniques [20]; for mold and yeast count using spread method [21] and for *Salmonella* [22] and *Shigella spp.* using ES ISO 6579 and ES ISO 21567 [23] and the values were compared with national standard and WHO guidelines for bottled drinking water.

### 2.2 Sampling

A total of 222 samples (45 carbonated and 177 uncarbonated bottled drinking waters) of 44 brands produced in various regions of Ethiopia were collected by trained professionals from commercial private bottling companies and retail stores. Five units of the same lot of production of bottled water were collected randomly to be analyzed as a single sample. They were transported to the laboratory in cool boxes within 24 hours. These polyethylene terephthalate bottles of uncarbonated and glass bottles of carbonated bottled water were stored at 4^o^C and conducted within 48 hours of collection. Each bottle was thoroughly mixed to have an even distribution of microorganisms, and then equal volume of the sample from each bottle of water was taken and mixed together in a sterile container to have a composite sample of the desired volume.

### 2.3 Laboratory Methods

All samples were tested for hethetrotrophic plate count, mould and yeast counts, total and, thermotolerant coliforms, *E. coli*, *S. aureus*, *Salmonellae and Shigellae* using accepted methodologies [18-21].

#### 2.3.1 Heterotrophic Plate Count

Heterotrophic plate counts were enumerated by pour plating a 1.0 mL volume of sample undiluted and in ten-fold dilutions using melted plate count agar and incubated for 48 ± 3 hours at 35^o^C. All colonies were counted as colony-forming units per milliliter [18].

#### 2.3.2 Enumeration of Yeasts and Moulds

Yeast and mould counts were performed according to ISO 21527-1:2014 using spread method on Rose Bengal chloramphenicol agar and incubated at 25^o^C for seven days [21].

#### 2.3.3 Coliform Assay

The total coliform count test was based on the multiple tube fermentation method to estimate the Most Probable Number (MPN) of total coliform, thermotolerant coliform and *E. coli* in 100 mL of water (ES ISO 9308-2:2001). *The* coliforms were estimated per 100 ml of water. The test was carried out by incubating for 48 hours at 37^o^C of measured quantities of sample water (50, 10, 1 mL) into tubes of double- and single-strength MacConkey broth (Difco). The tubes showing gas formation were considered to be presumptive coliform positive. The results of MPN was interpreted based on probability tables from the number of tubes showing acid and gas to estimate the number of the coliforms after confirmation with brilliant green lactose bile broth. Differential coliform count was done by incubating subcultures from the positive presumptive tests at 44^o^C in *E. coli* broth and at 44^o^C in tryptophan broth. The presence of coliforms was confirmed by the production of gas from lactose at 37^o^C, and that of *E. coli* was confirmed by the production of gas from lactose and indole from tryptophan at 44^o^C. *The* coliforms were estimated per 100 ml of water using MPN tables [20].

#### 2.3.4 Enumeration of *S. aureus*

*S. aureus* was performed by pouring plate method of ES ISO 6888-1:2018 using Baird-Parker agar medium incubated at 37^o^C for 24 hrs [19].

#### 2.3.5 Detections of Salmonella and Shigella Species

*Salmonella and Shigella spp* were performed by ES ISO 6579 and ES ISO 21567 using various broths followed by selective enrichment media and isolation media incubated at 37^o^C for 24 hours. Presumptive *Salmonella* and *Shigella* were subcultured on Xylose lysine deoxycholate agar and biochemically and serologically verified for confirmation [22, 23].

### 2.4 Data Analysis Procedures

The data was entered and analyzed using SPSS statistical package version 20.0. To reduce bias in the statistical analyses, values different from zeros (0.5) were used for results of no detections [24] The non parametric Kruskall-Wallis test was used to find out the differences in indicators values by regions, brands and analysis years and to observe the associations among the different organisms; the Spearman Rank Correlation was used because of non- normal distributed data and unequal size of the samples among the datasets. To test the normality of data and the existence of outliers, Shapiro-Wilk test was used. The significance level was set at p < 0.05. The findings of microbial analyses were compared with established Ethiopian standards and WHO guidelines of bottled drinking water.

## 3 RESULTS

### 3.1 Microbial Quality of Bottled Drinking Water

Among 222 bottled drinking water samples examined from 44 brands, detections of HPC, mold, yeast, total coliform, thermotolerant coliform, *E. coli* and *S. aureus* were observed in 114 (51.4%), 33 (14.9%), 5 (2.3%), 2 (0.9%), 1 (0.5%%), 1 (0.5%%) and 1 (0.5%%) samples respectively but there were no detection of *Salmonella* nor *Shigella* species. Bottled drinking waters contained heterotrophic plate counts of greater than 100 cfu/mL were noticed in 81 (36.5%) samples, 24 (50%) brands. Maximum counts, 1.0 × 10^4^cfu/mL to 4.8×10^5^cfu/mL HPC were observed in five brands; 1.2×10^3^cfu/mL to 1.2×10^4^cfu/mL for molds in three brands, 1.2 × 10^3^cfu/mL to 1.0×10^4^ cfu/mL for yeasts in three brands, 2.0×10^3^ MPN/100mL for total coliforms in one brand, 1.1×10^3^ MPN/100mL for thermotolerant coliform in one brand and 1.7×10^4^ cfu/g for *S. aureus* in one brand. The detections of heterotrophic bacteria and mold by regions were shown in Tables **1** and **2**.

The presence of molds was observed in 15 (34.1%) brands, Yeast in 3 (6.8%) brands, total coliforms in 2 (4.5%) brands, *S. aureus* in 1 (2.3%) brand, thermotolerant coliform and *E. coli* in 1 (2.3%) brand.

Out of 222 bottled drinking waters, 135 (60.8%) samples or 15 (34.1%) brands were free of yeast, mold, *S. Aureus, Shigellae* and *Salmonella* species and having HPC of less than 100 cfu/mL, but the rest 87 (39.2%) samples or 29 (65.9%) of the brands contained one or more of these microorganisms. Molds were detected in 6 (2.7%) of the samples in the absence of any coliforms, *S. aureus*, *Shigellae* and *Salmomnelae* species and in the accepted range of HPC; in 27 (12.2%) samples in the absence of any coliform, *Shigella* and *Salmomnelae* species.

The results of Spearman’s rank correlation coefficient r (significant at the 0.05 level, 2-tailed) of HPC, mold, yeast, total coliform, thermotolerant *Coliform*, and *S. Aureus * is shown in Table **3**.

Regionally, HPCs above 100 cfu/mL were observed in 13 of 25 (52%) bottled water samples and mold detected in 5 (20%) samples; Yeast, total coliforms, thermotolerant coliforms, *E. coli*, *S. aureus*, *Salmonella* and *Shigella* species in none of the samples in Addis Ababa city administration. HPCs above 100 cfu/ml were observed in 54 of 173 (31.2%) water samples and mould detected in 26 (15%) samples; yeast in 5 (2.9%) of the samples with counts of 1.5×10^2^, 1.2×10^3^, 1.0×10^4^, 1.8×10^2^ and 3.0×10^3^ cfu/mL; total coliforms in 2 (1.2%) samples; thermotolerant coliforms, *E. coli*, and *S. aureus* in 1 (0.6%) bottled water samples and *Salmonella* and *Shigella* species in none of the bottled samples in Oromia. HPCs above 100 cfu/mL were observed in 3 of 8 (37.5%) water samples and mold detected in 1 (12.5%) samples; yeast, total coliforms, thermotolerant coliforms, *E. coli*, *S. aureus*, *Salmonella* and *Shigella* species in none of the bottled water in Amhara region. HPC s above 100 cfu/mL were observed in 7 of 11 (63.6%) bottled water samples and mould, yeast, total coliforms, thermotolerant coliforms, *E. coli*, *S. aureus, Salmonella* and *Shigella* species in none of thee bottled water in Tigrai region. HPCs above 100 cfu/ml were observed in 4 of 4 (100%) bottled water samples and mould, yeast, total coliforms, thermotolerant coliforms, *E. coli*, *S. aureus*, *Salmonella* and *Shigella* species in none of bottled water in Dire Dawa city administration. One bottled water sample in Somali had no mold, yeast, total coliforms, thermotolerant coliforms, *E. coli*, *S. aureus, Salmonella and Shigella* species detection with HPC lower than 100 cfu/ml.

All the carbonated bottled drinking water samples were free from yeast, total and thermotolerant coliforms, *S. aureus*, *E. coli*, *Salmonella* and *Shigella*. However, heterotrophic plate counts greater than 100 cfu/mL were observed in 10 (22.2%) samples and detection of mold in 3 (6.6%) sample. P-value for heterotrophic plate count using Kruskall-Wallis test for total samples by carbondioxide treatment status was 0.007.

## 4 DISCUSSION

In the present study, microbiological quality and safety of bottled water was assessed from six regions of Ethiopia using HPC, moulds, yeasts, total and thermotolerant coliforms, *E. coli*, *S. aureus, Shigella* and *Salmonella* species. About 40% of the bottled drinking water samples contained one or more of the bacterial indicators, mould and yeasts above the national standards or WHO drinking water guideline. The bottled drinking waters had unacceptable detection limits of heterotrophic plate counts above 100 cfu/mL when incubated at 37^o^c in 36.5% and mould in 14.9%, yeast in 2.3%, total coliform in 0.9%, thermotolerant coliform, *E. coli* and *S. aureus* in 0.5% of the samples exceeding Ethiopian drinking-water standard CES 58: 2017 or WHO guideline value of “*no detections for bottled drinking water*” [25, 26].

The recovery of HPC above the acceptable limits in the current study was lower than other studies done by Kassenga, 2007 and Semerjian LA, 2011, who found 92% and 59.4% contaminations in bottled drinking water samples in Dar es Salaam, Tanzani [3] and Lebanon [27] respectively, and higher than the study by Abd El-Salam, 2008 in Egypt (30.9%) [28]. The maximum counts of HPC observed in this study were in line with the study done by Majumder, 2011 in Dhaka, Bangladesh [29]. Its presence in the bottled drinking water in high numbers may designate the necessity of treatment and maintenance of bottled drinking water systems [5] and the occurrence of potential pathogens and opportunistic bacteria that can cause infections, especially in immunocompromised ones [30]. The occurrence of HPC in high numbers may be due to the use of contaminated bottles or equipments during bottling and failure of ozonation or UV equipment or contamination of the water by workers [31].

The contamination of bottled drinking water with mould in this study was lower than reported in the study conducted in Brazil by Yamaguchi *et al*., 2007 who detected mould in 35% of total samples. Yamaguchi *et al*., 2007 also detected yeasts in 36.6% of the samples [32] which is much lower than the current study. However, in a study conducted in Egypt by El-Salam, 2008, there was no bottled water contamination by any fungi [28]. The presence of mould and yeast indicates a process safety and the level of quality control [33].

The incidence of total coliforms in 0.9%, thermotolerant coliforms and *E. coli in 0.5% of the samples* of bottled drinking water in the present study indicates the possible presence of pathogenic enteric microbes [34], inadequacy of water treatment [35], handling and purification process, washing and filling phase of the bottles [3] due to poor hygienic practices of the producers, poor hand hygiene, illiteracy and unhygienic practices of vendors [13]. Various studies conducted by Ahmed W *et al*., 2013 in Brazil [36] and Gangil R, 2013 in Jaipur city, India [35] reported a high rate of detections of total coliforms, thermotolerant coliforms and *E coli* in bottled drinking water. While in Vikarabad, Telangana, India, Rao *et al*., 2016 [37] and Ashish *et al*, 2014 in Delhi, India revealed all the coliforms undetectable.

The presence of other indicator organisms, *S. aureus* in one sample (0.5%) indicates poor hygienic practices during the bottling process since it is part of the commensal skin flora [38] and common environmental contaminants which may diminish the efficiency of treatment process [39] which introduced into bottled water by the individual involved in water processing [38]. However, the existence of *S. aureus* in bottled water used for infant food preparation should raise some concerns [9]. The detection of *S. aureus* in bottled drinking water is considerably high in studies carried out by Igbeneghu and Lamikanra, 2014 in south-western Nigeria [13] and Al-Zahrani and El-Hamshary, 2013 [40] in Alexandria when compared to the current finding.

Detection of *Shigella* spp. and *Salmonella* in bottled drinking water are a high risk for public health and indicate enteric contamination due to lack of personal hygiene during production [15]. Odeyemi, 2015 in Nigeria [15] have reported the detection of *Salmonella*; and Ahmed W, 2013 in Dhaka, Bangladesh [36] detected *Shigella* spp. in bottled drinking water contrary to the present study.

The contamination of bottled water by at least one microbe, HPC, moulds, yeasts, total and thermotolerant coliforms, *E. coli*, *S. aureus*, *Shigella* and *Salmonella* spp. in 39.2% of samples in the present study was two times less than the study investigated by Ahmed W, 2013 [36].

Spearman Correlation test signified statistically significant positive correlations between mould counts and presence of yeasts, total and thermotolerant coliforms; total and thermotolerant coliforms were also correlated to each other (Table **3**). The correlation between yeasts and total coliforms in the current study was inconsistent with the study done in Brazil and could indicate the same source of contamination of bottled drinking water by both microorganisms [32]. The lack of correlation between HPC enumeration and total coliforms, and thermotolerant coliforms in this study was agreed with the study done by Akond, 2009 in Bangladesh [41]. The nonparametric Kruskall-Wallis test indicated that HPC differed statistically by region, brand and years (Table **4**).

The detection of microbes in bottled water can originate from poor quality-water source or ineffectiveness of the treatment technique employed [42]. Quality and the safety of water are considerably improved by advanced water treatment methods [39]. In the present study, carbon dioxide and filtration were the major treatment methods employed to inactivate or remove microbes in the carbonated bottled water industry and ultraviolet, ozone and filtration for the non-carbonated water.

The absence of yeast, total and thermotolerant coliforms, *S. aureus*, *E. coli*, *Salmonella* and *Shigella* and the lower detection of HPC or mold in the carbonated bottled drinking water samples was may be due to toxic effect of carbon dioxide on microbes and the lack of additional nutrient from glass bottles for the growth of microorganisms unlike polyethylene terephthalate bottles [16, 43]. Heterotrophic plate count for total samples by carbon dioxide treatment status or bottle water type was statistically significant (P-value = 0.007). The carbonated water does not allow bacterial growth but their growth occurs in non-carbonated natural mineral waters a few days after filling and storage at room temperature [44].

## CONCLUSION

About 40% of the bottled water samples were contaminated by fungi or bacterial indicators exceeding the standards allowable limits. This indicates bottled drinking waters are polluted by personnel who involved in the bottling process, environmental and fecal contaminants which are threats for human health. The detection of total coliforms in two brands and *E. coli* in one brand of bottled water suggests problems in the production process and the presence of different pathogens which cause major public health hazards. Carbon dioxide is the best water treatment method over ultraviolet and ozone and glass bottles are the preferred water container over polyethylene terephthalate bottles for the quality and safety of water. To avoid risks associated with bottled water contaminations, regulatory bodies and bottling companies should take timely action.

## Figures and Tables

**Table 1:** Enumeration of total heterotrophic bacteria in bottled drinking waters in different regions of Ethiopia between January 2008 and December 2015.

**Regions**		**Heterotrophic Plate Count (cfu/mL)**					
	<1	1-100	101-300	301-500	501-1000	1001-480000	Total
**AA**	6	6	3	2	1	7	25
**Oromia**	99	20	9	8	4	33	173
**Amhara**	4	1	2	0	0	1	8
**Tigray**	4	0	1	2	1	3	11
**DD**	0	0	0	1	0	3	4
**Somali**	1	0	0	0	0	0	1
**Total**	114	27	15	13	6	47	222

**cfu**–colony forming unit, AA–Addis Ababa, DD–Dire Dawa

**Table 2:** Enumeration of mold in bottled drinking waters in different regions of Ethiopia between January 2008 and December 2015.

**Regions**		**Mold Count (cfu/ml)**					
	<1	1-100	101-300	301-500	501-1000	1001-480000	Total
**AA**	20	1	3	0	0	1	25
**Amhara**	7	0	1	0	0	0	8
**DD**	4	0	0	0	0	0	4
**Oromia**	147	2	9	9	2	4	173
**Somali**	0	0	1	0	0	0	1
**Tigray**	11	0	0	0	0	0	11
**Total**	189	3	14	9	2	5	222

Cfu–colony forming unit, AA–Addis Ababa, DD–Dire Dawa

**Table 3:** Rho values of HPC, mold, yeast, total coliform, thermotolerant coliform, and *S. Aureus, Shigellae* in bottled drinking water samples between January 2008 and December 2015.

Parameters	Mold	Yeast	HPC	Tc	TTC	*S. Aureus*
Mold	1				
Yeast	0.298	1				
HPC	0.029	0.055	1			
total coliform	0.165	0.268	-0.024	1		
thermotolerant coliform	0.174	0.447	0.099	0.274	1	
*S. Aureus*	-0.022	-0.010	-0.056	0.012	-0.005	1

P-values for HPC, mold, yeast, total coliform, thermotolerant coliform, and *S. aureus*, using non parametric Kruskall-Wallis test for drinking water samples by brand, years and region were indicated in Table **4**.

**Table 4:** p-values for HPC, mold, yeast, total coliform, thermotolerant coliform, and *S. aureus*, in drinking water samples by brand, years and region between January 2008 and December 2015.

**Parameters**	**Brand**	**Years**	**Regions**
**Mold**	0.448	0.544	0.177
**Yeast**	0.998	0.014	0.920
**HPC**	0.001	0.001	0.008
**total coliform**	0.999	0.087	0.964
**thermotolerant coliform**	0.998	0.003	0.998
